# Turning the Tide Against Regulatory T Cells

**DOI:** 10.3389/fonc.2019.00279

**Published:** 2019-04-16

**Authors:** SeongJun Han, Aras Toker, Zhe Qi Liu, Pamela S. Ohashi

**Affiliations:** ^1^Princess Margaret Cancer Centre, Campbell Family Institute for Breast Cancer Research, University Health Network, Toronto, ON, Canada; ^2^Department of Immunology, Faculty of Medicine, University of Toronto, Toronto, ON, Canada; ^3^Department of Medical Biophysics, Faculty of Medicine, University of Toronto, Toronto, ON, Canada

**Keywords:** immune regulation, Treg cells, T cells, tumor immunity, immune therapy

## Abstract

Regulatory T (Treg) cells play crucial roles in health and disease through their immunosuppressive properties against various immune cells. In this review we will focus on the inhibitory role of Treg cells in anti-tumor immunity. We outline how Treg cells restrict T cell function based on our understanding of T cell biology, and how we can shift the equilibrium against regulatory T cells. To date, numerous strategies have been proposed to limit the suppressive effects of Treg cells, including Treg cell neutralization, destabilizing Treg cells and rendering T cells resistant to Treg cells. Here, we focus on key mechanisms which render T cells resistant to the suppressive effects of Treg cells. Lastly, we also examine current limitations and caveats of overcoming the inhibitory activity of Treg cells, and briefly discuss the potential to target Treg cell resistance in the context of anti-tumor immunity.

## Introduction—Regulatory T Cell in Cancer

### Challenges in Immune-Oncology—Immunosuppressive Cells

The concept of utilizing the T cells, to recognize and eliminate cancer cells has contributed to the advancement of immunotherapy against multiple malignancies. Recent advances in checkpoint inhibitors (in particular CTLA-4 and PD-1 inhibitors) and cell-based therapy such as Chimeric Antigen Receptor (CAR)—T cell therapy demonstrate promising clinical responses in various cancer types in a subset of patients. However, despite the attempts to modulate anti-tumor T cell responses, a proportion of patients still do not respond to these immune therapies (1–3). The mechanisms of resistance against immune therapy is currently a key area of investigation. Some of these mechanisms include the presence of immunoregulatory cells in the tumor microenvironment such as tumor-associated macrophages (TAMs), myeloid-derived suppressor cells (MDSCs) and regulatory T (Treg) cells which could play an important role in restricting T cell immunity (4–6). Thus, overcoming the effects of these immunosuppressive cells remain a challenge for those seeking to enhance anti-tumor immune response.

### Evidence for a Role for Regulatory T Cells in Anti-tumor Immunity

Treg cells are one of the integral components of the adaptive immune system that contribute to maintaining tolerance to self-antigens and preventing autoimmune diseases (7, 8). It is postulated that these cells have an important role in regulating immune surveillance and promoting tumor progression. However, their precise role in regulating anti-tumor immunity and the mechanism of how Treg cells could suppress T cells in tumor is still unclear (9). Early studies used CD4^+^CD25^+^ markers to identify Treg cells with the caveat that activated helper T cells would also express these markers (10). Woo et al. (11) provided evidence for the presence of regulatory T in patients with early-stage non-small cell lung cancer and late-stage ovarian cancer. Numerous other manuscripts have also noted the presence of potential CD4^+^CD25^+^ Treg cells in multiple types of cancer including melanoma, pancreatic cancer and breast cancer (12–14).

In 2003, studies reported that the transcription factor FoxP3 was critical for Treg development (15–17), Subsequently, Curiel et al. (18) examined CD4^+^CD25^+^FoxP3^+^ cells and found that increased infiltration of Treg cells correlated with disease progression in ovarian carcinoma, and infiltration of these cells in each stage of cancer served as a good metric for survival prediction. Similarly, studies demonstrated that the presence of Treg cells in breast cancer correlated with reduced overall survival (19, 20). In contrast, several reports suggested that infiltration of Treg cells can be a favorable prognostic factor (21–24). Such discrepancies may result from the inability to precisely identify regulatory T cells within the heterogenous pool of FoxP3^+^ expressing CD4^+^ T cells (25). Alternatively, considering high infiltration of Treg cells also correlate with high infiltration of CD8^+^ T cells in a specific tumor subtypes (24), regulatory T cells may be recruited in response to an inflamed tumor microenvironment. Part of the controversy could also be due to the finding that FoxP3 can be transiently upregulated in activated human T cells, and is therefore not an exclusive marker for Treg cells (25, 26). The expression level of other markers such as CD45RA (27) and Treg-specific DNA demethylation status within the *FoxP3* locus can increase the accuracy of identifying functionally active Treg cells (28, 29). However, it is not always possible to perform these in depth analysis. Studies have also utilized *ex vivo* Treg suppression assays to demonstrate the presence of regulatory T cells within tumor tissue (18, 30, 31).

In mice, the role of Treg cells in regulating anti-tumor immunity has been investigated through ablation of Treg cells (using FoxP3^DTR^ mice or antibodies targeting receptors highly expressed on Treg cells, such as CD25, GITR, and folate receptor 4) in transplantable tumor models (32–35). In these models, depletion of regulatory T cells in conjunction with modulation of T cell immunity improves anti-tumor immunity. In contrast, co-adoptive transfer of CD8^+^ T cells with Treg cells prevented effective adoptive cell therapy against B16-F10 melanoma (36). In summary, although the presence of Treg cells in tumors cannot be used as an accurate prognostic factor, the literature suggests that Treg cells are a potent regulator of anti-tumor immunity.

### Immune Therapy and Treg Cells

One potential mechanism that may reduce the efficacy of cancer immunotherapy is suppression mediated by the Treg cell population. In addition, the therapeutic modalities such as anti-PD-1 may potentially alter Treg cell function and/or frequency, either directly or indirectly by changing the immune microenvironment (37–39). Thus, the potential effect of Treg cells on tumor-specific T cells should not be neglected even in therapeutic arena.

One of the most predominantly utilized checkpoint inhibitors in clinical and translational studies involve therapeutic blockade of PD-1 (nivolumab and pembrolizumab) or PDL-1 (atezolizumab and duravalumab) (40). There is a limited number of clinical studies thoroughly documenting changes in the quantity and quality of Treg cells in response to these PD-1/PD-L1 inhibitors. To date, studies either report an increase or no change in the frequency of Treg cells in response to nivolumab or pembrolizumab (39, 41). It is also important to note that PD-1 and PD-L1 can be expressed by Treg cells, thus direct modulation of Treg cell function should not be excluded as a possibility (31, 42–44). A few reports demonstrate that PD-1 blockade attenuates Treg cell suppression *in vitro*, based on the effect of PD-1 inhibitor on T cell proliferation in the presence of Treg cells (39, 45, 46). However, the effect of these inhibitors on Treg cells have not been clearly discriminated against its effect on T cells. A few reports including a study conducted by Toor et al. (47, 48) suggest that PD-1 blockade does not modulate Treg cell phenotype or function, but instead targets activated T cells. A murine study conducted by Chen et al. (49) demonstrates that PD-1 has no influence over the development and suppressive effects of thymically-derived Treg cells, however PD-1 appears to be crucial for differentiation of naïve CD4^+^ T cells into iTregs. Similarly, PD-L1 blockade can interfere with the induction and maintenance of iTreg cells in mice (50). Collectively, the precise effect of PD-1 blockade on Treg cells is poorly understood. Nevertheless, PD-1 inhibition synergizes with therapeutic strategies which reduce the quantity of Treg cells in mice (35, 51, 52), suggesting that enhanced anti-tumor immunity in response to PD-1 blockade may still be limited by Treg cells. Extensive studies have been performed evaluating the clinical potential of interfering with immune checkpoint receptors beyond PD-1, including CTLA-4, LAG-3, and TIM-3. However, the effect of each checkpoint inhibitors on Treg cells is also poorly understood and are beyond the scope of this review.

Adoptive cell therapies using TCR transduced T cells, CAR-T cells and Tumor-infiltrating Lymphocytes (TIL) are capable of directly recognizing and targeting tumor cells (3, 53). However, whether or not these T cell products are susceptible to regulation by Treg cells in humans is yet to be elucidated. In a few cases, the frequency of lymphocytes resembling Treg cells increases with adoptive T cell therapy (37, 38, 54). In the context of TIL therapy, Yao et al. (37) has demonstrated that the quantity of Treg cells reconstituted after non-myeloablative chemotherapy, which correlates with the number of administered doses of IL-2, is associated with patient responsiveness to TIL therapy. Supportive of this finding, administration of high-dose IL-2 (often utilized in conjunction with TIL therapy) can result in expansion of immunosuppressive ICOS^+^ Treg cells, which may be predictive of clinical outcomes in patients with metastatic melanoma (55). Baba et al. (56) utilized a murine model of fibrosarcoma to suggest that rapid reconstitution of Treg cells post-lymphodepletion suppress anti-tumor immunity, and targeting these regulatory T cells using neutralizing antibodies significantly reduced tumor growth. In the context of CAR-T cell therapy, the effect of the treatment on Treg cells may vary. For instance, clinical infusion of EGFRvIII-directed CAR-T cells for the treatment of glioblastoma resulted in influx of CD4^+^CD25^+^FoxP3^+^ cells in the tumor (38), whereas CD19-targeted CAR-T cells against B-cell lymphoma and leukemia did not increase the frequency of Treg cells (57). Lymphodepletion, known to transiently reduce the frequency of Treg cells, improves persistence of CAR-T cells as well as therapeutic outcome (58), however the direct effect of Treg cells on CAR-T cells is unknown. In summary, the role of regulatory T cells in the context of adoptive T cell therapy is currently unknown, however the literature suggests that Treg cells may limit the outcome of these therapeutic modalities.

### Mechanisms of Treg Suppression

The general mechanisms of T cell suppression by Treg cells, mostly evaluated through *in vitro* experiments, suggest that Treg cells may exploit diverse contact-dependent and cytokine-mediated mechanisms to limit T cell function (59, 60). One of the proposed mechanisms involve the ability of Treg cells to downregulate CD80/86 expression on dendritic cells (61–63). In a study conducted by Wing et al. (62, 64) and Onishi et al. (63), Treg-specific deletion of CTLA-4, which binds to CD80/86, results in reduced suppressive effects of Treg cells *in vivo* and failed to downregulate CD80/CD86 expression on dendritic cells (DCs) *in vitro*. Qureshi et al. (65) also demonstrate that CTLA-4 can reduce CD80/CD86 expression on DCs through trans-endocytosis and subsequent degradation of the co-stimulatory molecules. Furthermore, *in vitro* engagement of CTLA-4 with cognate receptors on DCs reduces the secretion of cytokines by DCs such as IL-6 and TNF, while increasing the expression of IDO, an immunosuppressive tryptophan catabolizing enzyme (66, 67). However, evidence also suggests that Treg cells can maintain suppressive functions without CTLA-4. For example, Paterson et al. (68) demonstrated that conditional ablation of CTLA-4 in adult mice do not result in systemic autoimmunity as observed in germline CTLA-4 deficiency, and also suggested that these Treg cells deficient in CTLA-4 are functional both *in vitro* and *in vivo*. Several other potential mechanisms of T cell suppression have been proposed, including (1) increased interaction between Treg cells and dendritic cells through high expression of LFA-1 on Treg cells resulting in reduced T cell priming (63, 69), (2) perforin and granzyme-mediated lysis of effector T cells (70–72), and (3) CD39 and CD73-mediated metabolic disruption of T cells (73). Through *in vitro* experiments, Deaglio et al. (73) suggested that CD39 and CD73 (ectonucleotidases used for hydrolysis of phosphate residues) expression by Treg cells can induce hydrolysis of extracellular ATP to adenosine, which triggers A2A receptor on T cells and elevates intra-cellular cAMP for T cell inhibition. However, most of these proposed mechanisms have not been explored *in vivo*.

Treg cells may also attenuate the T cell response via the production of chemokines and inhibitory cytokines. Treg cells can secrete TGF-β, IL-10, and IL-35 in a context-dependent manner, and reduce effector T cell function (74–77). For example, TGF-β can be a potent regulator of CTL function *in vitro* and *in vivo* (76, 78, 79), and reduce anti-tumor immunity in a transplantable tumor model (76, 79, 80). Although the secretion of TGF-β by Treg cells appears to be an important mechanism of suppression, an *in vitro* study conducted by Piccirillo et al. (81) also suggests that blockade of TGF-β produced by regulatory T cells do not reduce the suppressive effects of Treg cells. The role of IL-10 on T cells is unclear due to evidence of IL-10 serving as either stimulatory or inhibitory cytokine in a context-dependent manner, however evidence suggests that IL-10 plays an important role in Treg cell-mediated suppression of T cells (82, 83). For instance, Chaudhry et al. (82) suggests that IL-10 signaling acts on Treg cells to attenuate pathogenic T_h_17 response, however, the molecular mechanism of T cell suppression is still unclear. Similarly, the precise mechanism of T cell inhibition by IL-35 is also unclear, but studies suggest that IL-35 restricts T cell proliferation and induces “infectious tolerance” by inducing Treg cells from naïve CD4^+^ T cells (84, 85). Lastly, in conjunction with previously described cytokine-driven suppressive mechanisms, it has been recently demonstrated in EAE and islet allograft models that secretion of the chemokines CCL3 and CCL4 by Treg cells plays an important role in the recruitment of effector T cells to close proximity of Treg cells where they become susceptible to suppression (86).

Lastly, *in vitro* Treg suppression assays suggest that Treg cells compete with other T cells for IL-2, and that the decreased availability of IL-2 reduces T cell proliferation and function (87–89). In this particular system, Treg cells constitutively express a high level of high-affinity IL-2 receptors whereas stimulated naïve T cells do not express high-affinity IL-2 receptors at an earlier time point; this may further contribute to preferential acquisition of IL-2 by Treg cells. Furthermore, IL-2 provides STAT5 signaling in Treg cells that is necessary to further enhance their immunosuppressive function (90, 91). This particular mechanism of suppression can also be observed *in vivo*. A study conducted by Chinen et al. (91) suggest that the ability of Treg cells to capture and compete for IL-2 is critical for controlling CD8^+^ T cell expansion and function. The general consensus for those investigating Treg cell-mediated suppression of T cells is that each suppressive mechanism likely acts in a context-dependent manner and more than one mechanism could be employed simultaneously to inhibit T cell function (7, 59). Thus, the ability of Treg cells to compete for IL-2 likely works in tandem with other suppressive mechanisms to regulate T cell immunity.

It remains unclear which of the previously described mechanisms are relevant for regulatory T cells residing in the tumor. Treg cells found in the tumor often display a distinct phenotype in comparison to those circulating the periphery, which is exemplified through their unique transcriptional signatures and the expression of markers including PD-1 (31, 43, 44, 92, 93). In the context of head and neck squamous cell carcinoma, tumor-infiltrating CD4^+^CD25^hi^Foxp3^+^ T cells produce a higher level of TGF-β and reduced T cell proliferation more effectively than Treg cells from the periphery in Treg suppression assays (30, 94). These correlative studies suggest that intra-tumoral Treg cells display highly immunosuppressive phenotype *in vitro*, suggesting that they may regulate anti-tumor immunity. However, it is still unclear precisely “when,” “where” and “how” these distinct Treg cells exert their suppressive effect in cancer biology. Most *in vivo* and *in vitro* experiments performed to elucidate the cellular and molecular mechanism of T cell suppression by Treg cells in mice were performed using Treg cells from secondary lymphoid organs such as spleen and lymph nodes, and therefore may not fully recapitulate the interaction between intra-tumoral Treg cells and T cells. Nevertheless, evidence acquired from studies using non-tumor derived Treg cells may provide insights in understanding how intra-tumoral Treg cells could potentially limit anti-tumor T cells.

### Potential Strategies to Interfere With Immune Suppression by Regulatory T Cells

**Figure 1:**
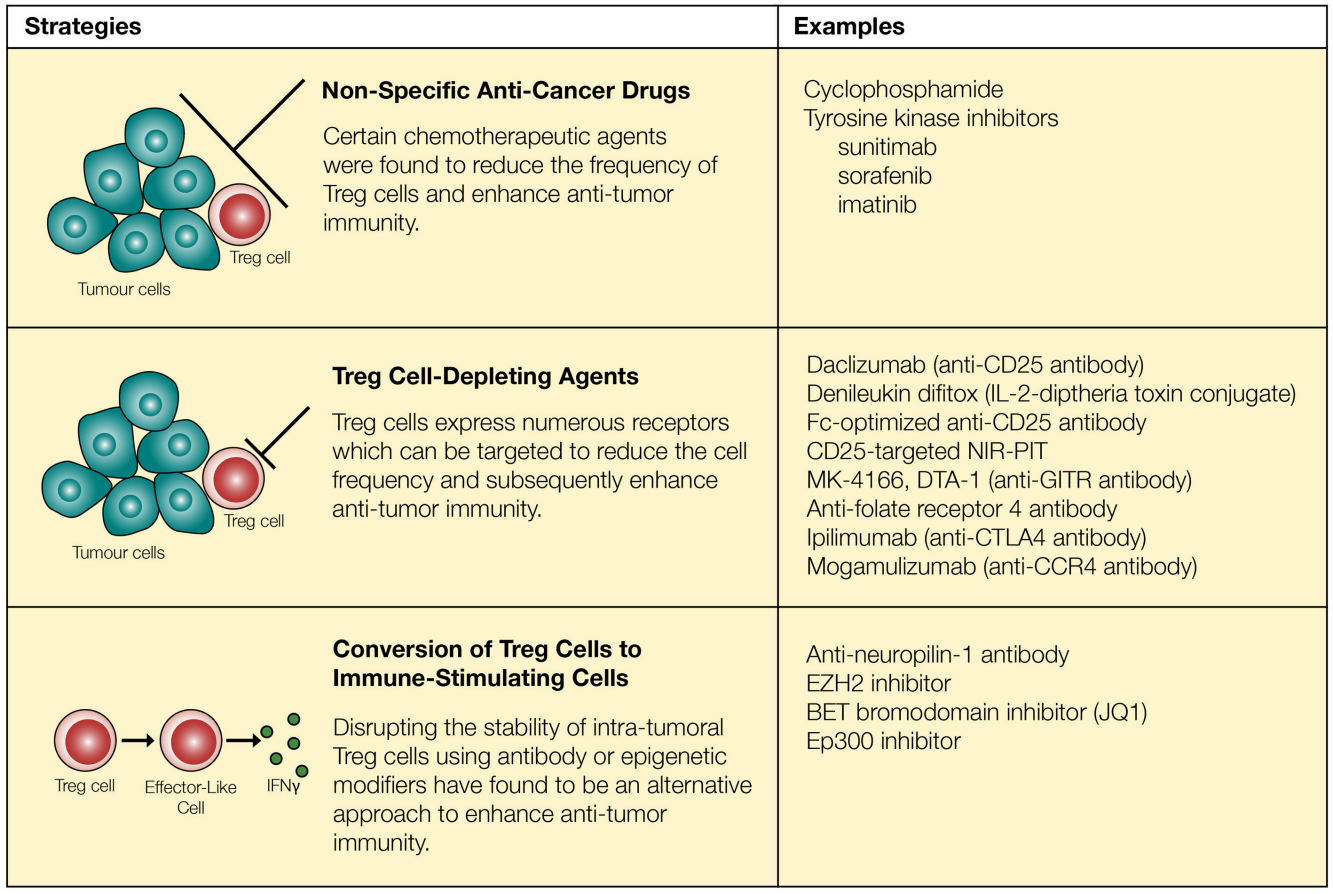
Regulatory T cells can be targeted through chemotherapeutic agents, neutralizing antibodies and epigenetic modifiers. Chemotherapeutic agents reduce the quantity of Treg cells and synergize with immune-modulatory drugs to enhance anti-tumor immunity. However, these approaches are not necessarily specific to Treg cells. Surface markers expressed on Treg cells (such as CD25, GITR, folate receptor 4, CTLA-4, and CCR4) can be targeted to more reliably reduce the quantity of Treg cells. Recent approaches involve conversion of Treg cells into “effector-like” CD4^+^ T cells through the use of neutralizing antibodies as well as epigenetic modifiers.

Acknowledging the significance of Treg cells and their potential role in inhibiting anti-tumor immunity, multiple strategies have been proposed to deplete Treg cells *in vivo*. However, one major challenge associated with Treg cell depletion is the lack of a Treg cell-specific marker. Most surface molecules expressed on Treg cells are also present on activated T cells, although the level of expression may be different. Similarly, FoxP3 is expressed by both activated T cells and Treg cells in humans (25, 26). Despite such challenges, several potential strategies have been proposed to reduce the suppressive effects of Treg cells (Figure 1). First, several non-specific anti-cancer drugs have been shown to reduce Treg cell activities. Low-dose cyclophosphamide (CTX), a common chemotherapeutic agent known to target rapidly dividing cells, significantly reduced Treg cells owing to their higher rate of proliferation, leading to enhanced anti-tumor immunity (95–98). In these studies, investigators have noted that CTX reduced the levels of intra-tumoral Treg cells while maintaining or elevating the level of CD8^+^ T cells in the tumor (96, 97). In contrast, several studies have reported contradicting data where CTX either increased the level of Treg cells or did not enhance anti-tumor immunity (99, 100). Additional studies showed that treatment with CTX was further improved in its selectivity and efficacy through combination therapy with OX40 agonist or anti-PD-1, demonstrating increased intra-tumoral Teff/Treg cell ratio and subsequent regression of B16 and TC-1 tumors (101, 102). Several other FDA-approved anti-cancer agents including tyrosine kinase inhibitors sunitinib, sorafenib, and imatinib also reduced the levels of intra-tumoral Treg cells (101, 103–105).

While specific targeting of tumor-infiltrating Treg cells can be challenging, several agents including daclizumab (CD25 blocking antibody), denileukin diftitox (Ontak, IL-2-diphtheria toxin conjugate protein), and several other antibodies have been proposed to target Treg cells and enhance anti-tumor immunity (106, 107) (Figure 1). First, the use of CD25 to target and deplete Treg cells has resulted in improved anti-tumor immunity in some cases (108, 109). However, this strategy has raised a number of concerns based on inconsistent *in vivo* responses and lack of specificity. Similar to the effects of anti-CD25 in mice (clone PC-61), the use of denileukin diftitox for depleting Treg cells and eliciting a stronger anti-tumor immune response remains controversial, due to varying clinical responses (110, 111). For instance, treatment of patients with renal cell carcinoma using denileukin diftitox effectively relieved inhibition by Treg cells to promote anti-tumor immunity, but the opposite trend was observed in patients with metastatic melanoma (110, 111). Tumor heterogeneity, the existence of CD25^−^ Treg cells and CD25 expression on other immune cells, such as T cells, B cells, and NK cells (112, 113), may explain seemingly opposite outcomes in this particular approach. However, recent studies have further modified and improved strategies targeting CD25 and suggest that it may still be a viable option to restrict Treg cell activities. Arce Vargas et al. (35) demonstrated that Fc-optimized antibodies against CD25 could effectively reduce the frequency of intra-tumoral Treg cells and improve tumor control. Furthermore, CD25-targeted near-infrared photoimmunotherapy (NIR-PIT) has been developed in a murine model. By conjugating anti-CD25 with a photoactivatable silica-phthalocyanine dye sensitive to near-infrared light, and localizing near-infrared irradiation specifically on tumors, NIR-PIT achieved reduction of intra-tumoral Treg cells (114).

Beyond CD25 as a target molecule, regulatory T cells constitutively express receptors such as GITR, CTLA-4, and folate receptor 4. In the tumor microenvironment, Treg cells further upregulate a large number of receptors including ICOS, OX40, GITR, TIGIT, PD-1, and CTLA-4 (31, 115). Antibodies targeting some of these receptors expressed by Treg cells such as GITR and folate receptor 4 reduce the amount of Treg cells and enhance anti-tumor immunity in mice (32, 33, 116). Similarly, checkpoint inhibitors designed to block inhibitory signals on T cells may also play an important role in regulating Treg cell activities. With Treg cells expressing a high level of CTLA-4 (27), administration of an anti-CTLA-4 antibody has resulted in a major reduction in the frequency of intra-tumoral CTLA-4^+^FoxP3^+^ Treg cells which was dependent on Fcγ receptor-expressing cells in the tumor microenvironment (117–121). This is consistent with the correlation of decreased frequency of tumor-infiltrating Treg cells with the usage of ipilimumab in patients with bladder cancer and advanced melanoma (122–124). Lastly, a study conducted by Sugiyama et al. (125) demonstrated that a high proportion of Treg cells express CCR4 in tumor-infiltrating lymphocytes (TILs) acquired from melanoma patients. CCR4 expression was specific to CD4^+^CD45RA^−^FoxP3^hi^ Treg cells, a terminally differentiated and highly suppressive subset of Treg cells that preferentially accumulates within tumors, whereas CCR4 is not expressed on CD4^+^CD45RA^+^FoxP3^lo^ naïve T cells. In agreement with these findings, administration of anti-CCR4 (Mogamulizumab) in patients with Adult T-Cell Leukemia-Lymphoma (expressing NY-ESO-1) resulted in reduction in CD4^+^CD45RA^−^FoxP3^hi^ Treg cells and enhanced NY-ESO-1-specific CD8^+^ T cell response (125). Although anti-CCR4 antibodies target a specific subset of Treg cells that are highly abundant within tumors, this particular strategy does not selectively deplete intra-tumoral Treg cells since a large proportion of Treg cells in peripheral blood are CD4^+^CD45RA^−^CCR4^+^FoxP3^+^ Treg cells (8, 27, 125).

Interestingly, studies published within the last few years suggest that promoting the conversion of Treg cells into immune-stimulatory cells could be an alternative approach to enhancing anti-tumor immunity (Figure 1). FoxP3^+^ regulatory T cells are comprised of heterogenous sub-populations of cells some of which display functional plasticity. Depending on the environmental cues, these Treg cells remain uncommitted and become susceptible to being re-programmed to FoxP3^−^ helper T cells or FoxP3^+^ cells which display properties of a helper T cell (126–129). Similarly, there are heterogenous populations of highly suppressive Treg cells in the tumor microenvironment. Although the composition and function of these tumor-infiltrating Treg cells is still a topic of debate, evidence suggest that both thymically-derived natural Treg cells, characterized by high expression of neuropilin-1, and induced Treg cells play important role in regulating anti-tumor immunity (130). Peripherally-derived regulatory T cells, which display greater plasticity, can be targeted to enhance anti-tumor immunity (130, 131). Furthermore, despite the initial assumption that thymically derived Treg cells undergo a strict lineage commitment, Overacre-Delgoffe et al. (132) demonstrated that targeting neuropilin-1 on Treg cells induces IFNγ production and “functional fragility” which can in turn enhance anti-tumor immunity. A recent approach of converting Treg cells into immune-stimulatory cells in the context of tumor immunity involve epigenetic modification of intra-tumoral Treg cells to disrupt their lineage and functional stability. For example, Wang et al. (133) have demonstrated that the histone H3K27 methyltransferase enhancer of zeste homolog 2 (EZH2) activities are increased in tumor-infiltrating Treg cells in both murine and human cancers, and molecular targeting of EZH2 promoted conversion of Treg cells into IFNγ producing cells that were capable of remodeling the tumor microenvironment and enhancing anti-tumor immunity. Several other epigenetic modifiers such as Bromodomain and Extra-Terminal (BET) family proteins and histone acetyltransferase Ep300 can also be targeted to disrupt Treg cell function and improve anti-tumor immune response (134, 135). However, these epigenetic modifiers possess other biological functions, and molecular targeting of these proteins could potentially induce off-target effects.

Despite these alternative approaches to Treg cell blocking or depletion strategies, limitations still exist, including the lack of a Treg cell-specific biomarker and potential induction of autoimmunity as a consequence of systemic Treg cell depletion (136, 137). Lastly, depletion of Treg cells can be followed by their rapid reconstitution, often resulting in a higher frequency in comparison to the level of Treg cells prior to depletion (138, 139). Alternatively, another approach to enhance anti-tumor immunity would be to modify tumor-specific T cells to be resistant to the suppressive effects of Treg cells. This approach may be relevant when adoptive T cell therapies are used including TCR transduction with tumor specific TCR or CAR-T cells.

## Reported Cases of Treg Resistance

**Figure 2:**
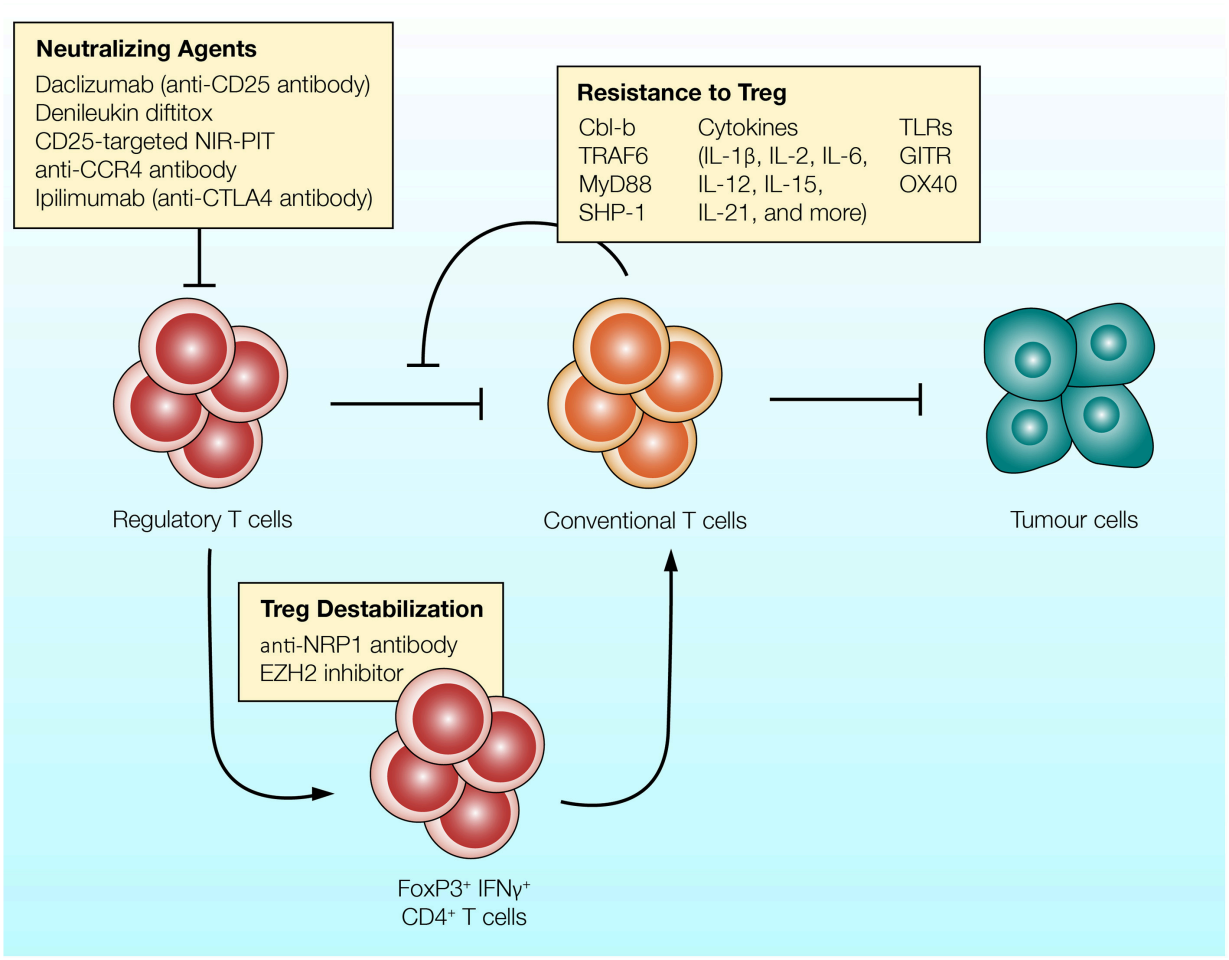
Different strategies can be utilized to overcome the suppressive effects of Treg cells. (1) Antibodies targeting CD25, CCR4, or CTLA-4 expressed on Treg cells can be used to reduce the frequency of regulatory T cells and enhance anti-tumor immunity. (2) Regulatory T cells can convert into T cell stimulatory cells in response to inhibition of EZH2 epigenetic modifier or NRP-1-targeting antibody. Treg cells treated with these agents upregulate IFNγ and enhance anti-tumor immunity (132, 133). (3) T cells can be rendered resistant to the suppressive effects of Treg cells. Intracellular molecules which govern T cell activation (such as Cbl-b and TRAF-6), co-stimulatory receptors (such as TLRs and GITR) and various T cell stimulatory cytokines reduce the ability of Treg cells to suppress T cells.

Since the early 2000s, evidence suggests that there are a variety of molecular pathways and cellular mechanisms which render T cells resistant to the suppressive effects of Treg cells. Numerous surface receptors, intracellular signaling molecules and cytokines have been implicated in T cell resistance to Treg cells (Figure 2).

### Intracellular and Receptor Targets Controlling Treg Resistance

#### E3 Ubiquitin Ligase Cbl-b

**Figure 3:**
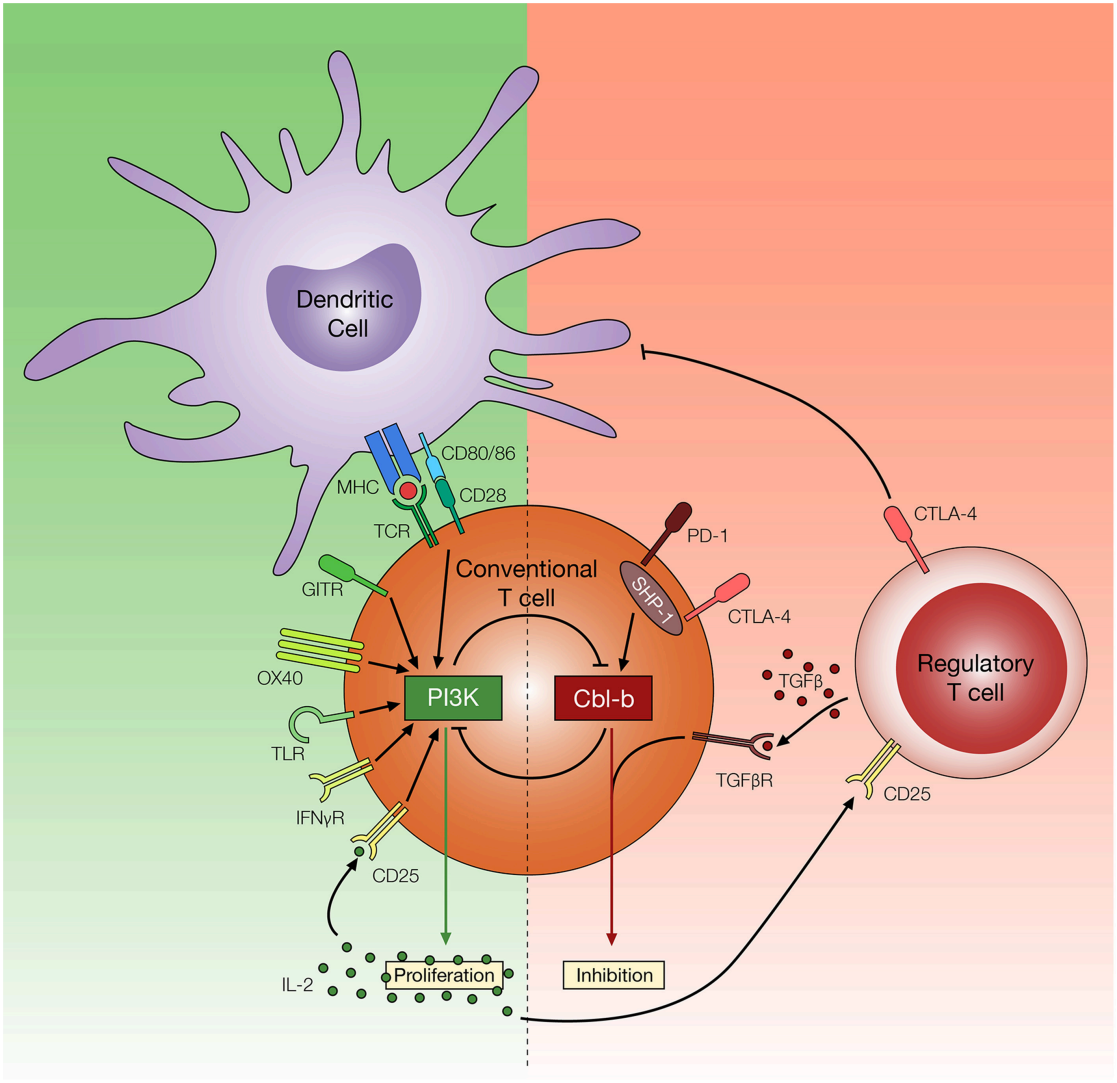
Potential mechanisms of T cell resistance to Treg cells. Regulatory T cells utilize multiple inhibitory mechanisms to limit T cell activation and proliferation, such as downregulation of CD80/86 on DCs, secretion of TGF-β, and consumption of IL-2. Reports suggest amplified PI3K signaling, through TCR, co-stimulatory and cytokine receptors, may render T cells resistant to these effects of Treg cells. In contrast, Cbl-b plays an important role in regulating diverse arms of the TCR signaling pathways and promoting T cell inhibition. Cbl-b deficient T cells are refractory to Treg cell-mediated suppression, but the mechanism of Treg cell resistance remains yet to be elucidated.

The inhibition of E3 ubiquitin ligase Cbl-b has shown promising results based on the ability of T cells to resist the suppressive effects of Treg cells both *in vitro* and *in vivo* (140, 141). Through ubiquitination (and in many cases, subsequent ubiquitin-mediated degradation) or phosphorylation of proteins involved in the TCR signaling pathway, Cbl-b serves as a negative regulator of antigen-induced T cell activation (142). Several molecular targets have been identified, including PKCθ, Nedd4, PLC-γ1, Vav1, LAT, and p85, along with several other TCR signaling molecules that play an important role in T cell activation (143–147). Consequently, through the regulation of these molecules, Cbl-b can control a diverse repertoire of intracellular mechanisms associated with the early phase of T cell activation, such as calcium influx, cytoskeletal rearrangement, immune synapse formation, cytokine secretion as well as proliferation (148, 149). Amongst several signaling pathways downstream of TCR activation, reports highlight the role of PI3K/Akt signaling pathway in T cell resistance to Treg cell-mediated suppression (150, 151). Interestingly, it has become evident that that PI3K and Cbl-b are indirectly regulated by each other to control T proliferation (Figure 3). Fang et al. (143) has suggested that Cbl-b regulates the PI3K signaling pathway by binding and ubiquitinating a PI3K regulatory subunit p85. However, a study conducted by Guo et al. (146) offers an alternative explanation where Cbl-b does not directly inhibit PI3K, but instead inhibits the Nedd4-mediated ubiquitination of PTEN, a negative regulator of PI3K activity. Adding to the complexity of the interaction between PI3K/Akt pathway and Cbl-b, Akt also negatively regulates Cbl-b protein level through inactivation of GSK-3, a protein kinase which enhances Cbl-b activity by catalyzing the phosphorylation at Ser476 and Ser480 (152).

In addition to the ability of Cbl-b to regulate molecular pathways associated with TCR signaling, evidence suggests Cbl-b is intertwined with multiple T cell inhibitory signaling pathways. Early studies demonstrated that Cbl-b can be re-expressed in response to CTLA-4 signaling, and CTLA-4 deficient T cells display reduced Cbl-b expression (153). Recent studies suggest that T cells deficient in Cbl-b are less susceptible to PD-1 inhibitory signaling *in vitro* (154, 155). These findings are consistent with a study suggesting that SHP-1, which plays an important role in downstream PD-1 and CTLA-4 signaling pathway, controls Cbl-b activity through direct phosphorylation (156). Furthermore, a study conducted by Mercadante and Lorenz (157) utilizes an *in vitro* Treg suppression assay and homeostatic *in vivo* Treg suppression assay to demonstrate that SHP-1 deficient T cells are less responsive to the suppressive effects of Treg cells. These studies suggest that Cbl-b is linked with key negative regulatory pathways in T cells. Lastly, Cbl-b is also intertwined with TGF-β receptor signaling. Gruber et al. (158) demonstrated that Cbl-b directly ubiquitinates and subsequently downregulates SMAD7, an attenuator of TGF-β receptor signaling. Consistent with this finding, CD4^+^ T cells deficient in Cbl-b display reduced sensitivity to TGF-β mediated inhibition (140, 141, 158, 159). The multi-faceted role of Cbl-b in regulating TCR signaling pathways as well the inhibitory signaling pathway enables Cbl-b deficient T cells to acquire TCR sensitivity, CD28-independent stimulation, increased cytokine production, and context-dependent TGF-β insensitivity (141, 160), all of which potentially contribute to T cell resistance to Treg cell-mediated suppression (Figure 3).

Cbl-b deficient CD4^+^ and CD8^+^ T cells resist Treg cell-mediated suppression in an *in vitro* Treg suppression assay, where naïve Cbl-b^−/−^ T cells stimulated with anti-CD3 and irradiated APCs are capable of overcoming the suppressive effects of splenic Treg cells (140, 161). However, (1) the ability of Cbl-b^−/−^ T cells to resist potentially “activated” Treg cells (such as those found in tumors) has not been explored, and (2) *in vitro* Treg suppression assay cannot recapitulate the complex interaction between T cells and Treg cells *in vivo* (60), especially since the Cbl-b^−/−^ mice do not have the same phenotype as Treg deficient mice (17, 162–164). Despite these limitations, many of the *in vitro* observations have been consistent with *in vivo* properties of Cbl-b^−/−^ T cells. For example, T cells deficient in Cbl-b also display a hyperactive T cell status *in vivo*. Gronski et al. (165) has demonstrated the role of Cbl-b in regulating T cell activation threshold, as mice deficient in Cbl-b were more sensitive to antigen-induced T cell stimulation resulting in autoimmunity. Lastly, Adams et al. (141) has demonstrated the role of Cbl-b in CD4^+^ T cell resistance to Treg cells *in vivo* through a graft-vs.-host disease model, where adoptively transferred Treg cells fail to suppress Cbl-b^−/−^ CD4^+^ T cells *in vivo*. However, the mechanism by which Cbl-b^−/−^ T cells resist Treg cell suppression has not been investigated in these studies.

T cells deficient in Cbl-b have also been studied in the context of enhancing tumor immune surveillance and anti-tumor immunity. Cbl-b deficiency augments anti-tumor T cell responses in both genetically engineered and transplanted tumor models (161, 166–168). Loeser et al. (161) and Chiang et al. (166) provide evidence showing a greater infiltration of CD8^+^ T cells using TC-1 and EL4/EG7 transplantable tumors in Cbl-b deficient mice. In both circumstances, CD4^+^ effector T cell infiltration did not increase. Interestingly, despite the increased infiltration of Treg cells in the tumors from Cbl-b deficient mice, T cells were able to either reject or attenuate tumor growth. A similar observation has been made when Cbl-b deficient mice were crossed with ataxia telangiectasia mutated (ATM) deficient mice, which attenuated the spontaneous development of lymphoid tumors and increased overall survival, demonstrating a robust anti-tumor immunity against genetically engineered tumor model (166). Although further investigation is required to understand how Cbl-b deficient T cells enhance anti-tumor immunity, one of the proposed mechanisms include insensitivity to TGF-β receptor signaling. Gruber et al. (158) suggested that Cbl-b deficiency promotes spontaneous rejection of TC-1 tumors, whereas Cbl-b^−/−^ mice crossed with CD4^Cre^- SMAD7^fl/fl^ mice abrogates anti-tumor immunity, thus highlighting the importance of Cbl-b deficient T cells in anti-tumor immunity and the ability of these T cells to potentially overcome TGF-β receptor signaling. Lastly, in all of the previously described studies, whether Cbl-b deficient T cells resist the suppressive effects of Treg cells to enhance anti-tumor immunity has not been shown *in vivo*.

#### TLR—MyD88—TRAF6 Axis

Evidence suggests that TLR signaling also play an important role in T cell resistance to Treg cells. Pasare and Medzhitov (169) suggested that TLR4 and TLR9-mediated stimulation of DCs and the subsequent increase in IL-6 production by DCs render T cells resistant to the effects of Treg cells. However, this particular study presumed that TLR signaling was restricted to DCs. TLRs can be expressed by effector T cells and Treg cells, and play an important role in their cellular activation and survival (170, 171). Although our understanding of TLR signaling pathways in T cells is rather limited, TLRs expressed on T cells likely function similar to co-stimulatory receptors which trigger the downstream MyD88 signaling pathway as well as the PI3K/Akt signaling pathway (172). TLR signaling in T cells may also play an important role in rendering T cells refractory to Treg cell-mediated suppression. For example, TLR9 stimulation of murine T cells enhances the PI3K/Akt signaling pathway and MyD88-dependent IL-2 production; TLR9 signaling also renders T cells resistant to the suppressive effects of Treg cells (173, 174). Downstream of TLRs, MyD88 interacts with IRAK1 and IRAK4, modulating the activities of an E3 ubiquitin ligase TRAF6 which may contribute to NFκB signaling (175). However, the role of TRAF6 in T cells is far more complex and contradictory, which is exemplified through a study suggesting that TRAF6 also serves as a negative regulator of T cell function (176). In this study, T cells deficient in TRAF6 display enhanced T cell activation, CD28-indpendent stimulation and resistance to Treg cell-mediated suppression (176). Although TLR signaling can promote T cell resistance to Treg cells, the precise molecular mechanism remains yet to be elucidated. It is worth noting that TLR stimulation of T cells increases cytokine production (173, 177), thus future studies should delineate the effect of TLR-MyD88 signaling vs. subsequently induced cytokines in generating resistance to Treg cells. Lastly, it is also crucial to evaluate the effect of TLR signaling on regulatory T cells which also express TLRs (170). The role of TLR signaling on Treg cell function requires further investigation and clarification since it can both abrogate and enhance Treg cell functions (170, 177–179). A recent study suggested that TLR signaling on regulatory T cells induces PI3K/Akt/mTORC1 signaling which subsequently increases glycolysis and GLUT1 expression, which in turn interferes with FoxP3 expression and the suppressive ability of Treg cells (180). However, increased Treg cell function observed in several studies could also occur indirectly as a result of enhanced T cell stimulation and IL-2 secretion, which can subsequently promote Treg cell function.

Although TLR agonists can improve anti-tumor immune responses by enhancing T cell function and/or stimulating APC maturation, they may also act on other immune cells and cancer cells to impact anti-tumor immunity (181, 182). Therefore, it would be difficult to specifically target TLRs to promote resistance to Treg cells.

#### TNF Family Members

TNF family members such as GITR, OX40, and 4-1BB on T cells can also be targeted to induce T cell resistance to Treg cells (183–188). Evidence suggests that amplification of GITR signaling through the use of agonistic antibody, DTA-1, enhances T cell stimulation in the presence of Treg cells both *in vitro* and *in vivo* (184, 189, 190). However, GITR is also highly expressed on Treg cells and studies suggests that a GITR agonist attenuates Treg cell stability (191, 192). In contrast, *in vivo* administration of non-depleting Fc-GITR-L induces context-dependent modulation of Treg cell activities (193). Further work is required to precisely understand the effect of GITR signaling on Treg cells. Although the role of GITR agonist in the interaction between T cell and Treg cell is unclear *in vivo*, Stephens et al. (184) suggested that GITR signaling directly acts on T cells to resist the suppressive effects of Treg cells *in vitro*. Lastly, a GITR agonist antibody (DTA-1) has demonstrated its potential in enhancing CD8^+^ T cell response and reducing intra-tumoral Treg cell activities using transplantable tumor models including the B16 melanoma model (190, 192, 194). In summary, administration of TNF-family receptor agonists such as those targeting GITR promote T cell response in the presence of Treg cells and contribute to enhanced anti-tumor immunity. However, the mechanism behind how TNF family receptor signaling renders T cells refractory to Treg cell-mediated suppression is poorly understood.

### Cytokine Networks

Most intracellular molecules and surface receptor targets which render T cells resistant to inhibition by Treg cells often promote the secretion of a high quantity of T cell stimulatory cytokines. This is demonstrated by the early study conducted by Pasare and Medzhitov (169), which showed that LPS stimulation of DCs leads to increased IL-6 which plays an important role in T cell resistance to regulatory T cells (169, 195). Similarly, inhibition of Cbl-b or activation of GITR signaling increases IL-2 production by T cells both *in vitro* and *in vivo* (167, 168, 183). Increased cytokine production is often perceived as an indicator of Treg resistance. However, evidence suggests that various cytokines themselves can directly drive T cell resistance to Treg cells (195–199). This raises a question—to what extent do cytokines play a role in Treg resistance? Both T cells and Treg cells are susceptible to cytokine receptor-mediated signaling, and therefore the effect of cytokines in both cell compartment must be considered.

Soluble mediators such as cytokines can modulate a powerful receptor-mediated T cell signaling required for cellular proliferation, survival, and resistance to Treg cell-mediated suppression. Cytokines including interferons (IFNγ and IFNα), those binding to receptors that include the common γ-chain (IL-2, IL-4, IL-7, IL-15, IL-21, and TSLP), gp130 receptor cytokines (IL-6) and IL-1 receptor cytokines (IL-1β and IL-18) employ diverse combinations of intracellular signaling pathways such as the JAK/STAT signaling pathways to promote T cell differentiation and effector functions (200–202). Many studies have also highlighted the role of T cell stimulatory cytokines, in particular IL-1β, IL-2, IL-4, IL-6, IL-7, IL-15, and IL-21, as central drivers of T cell stimulation in the presence of Treg cells (87, 195–198, 203–205). Some of these T cell stimulatory cytokines may induce T cell proliferation and survival in the presence of Treg cells by common mechanisms, because their receptors share overlapping downstream signaling pathways, but the mechanism by which each of these cytokines support T cell proliferation in the co-cultures has not been fully clarified.

One of the first cytokines reported to enhance T cell proliferation in the presence of Treg cells *in vitro* is IL-2 (199). Upon high-affinity quaternary IL-2-IL2R complex formation, tyrosine kinases JAK1, and JAK3 also initiate a STAT1, STAT3, and STAT5-dependent response, along with the induction of the PI3K signaling pathway (201, 202). Although IL-2 serves as a potent inducer of T cell proliferation in Treg suppression assays, there is no strong evidence suggesting that the signaling pathways downstream of IL-2 directly attenuates the inhibitory signals induced by Treg cells. Instead, excess IL-2 could enable T cells to overcome Treg cell-mediated cytokine deprivation (87, 199), which, despite being somewhat controversial, may be an important suppressive mechanism utilized by Treg cells (89, 91). Lastly, many T cell stimulatory cytokines including IL-2, IL-7, and IL-15 play an important role in enhancing anti-tumor immunity (206–208), but whether or not these cytokines render T cells resistant to the suppressive effects of Treg cells in the context of anti-tumor immunity is unclear.

When evaluating the role of cytokines in rendering T cells resistant to Treg cells, the effect of cytokine signaling must also be evaluated on Treg cells. Under a circumstance where T cell stimulatory cytokine destabilizes Treg cell function, it becomes challenging to determine whether T cell resistance to Treg cells play an important role in the observed T cell proliferation in the presence of Treg cells. Although poorly understood, Treg cells display phenotypic and functional plasticity in response to certain cytokines; T cell stimulatory cytokines may mediate the downregulation of FoxP3 or conversion of Treg cells into conventional T cells (209, 210). This is exemplified through a study which demonstrates the ability of IL-4 to convert FoxP3^+^ cells into effector CD4^+^ T cells, thereby undermining oral tolerance (211). PI3K signaling pathway is regulated by PTEN expression in Treg cells to prevent loss of Treg cell stability (212, 213), however, IL-4 may disrupt this process by enhancing PI3K signaling. Several other cytokines including IL-21 also antagonize Treg cell proliferation and reduce the frequency of Treg cells (214). However, a study conducted by Attridge et al. (215) suggest that IL-21 may act on T cells to limit IL-2 production which subsequently impairs Treg cell homeostasis. Furthermore, a recent study conducted by Overacre-Delgoffe et al. (132) suggests that attenuating Nrp-1 signaling on intra-tumoral Treg cells induces increased secretion of IFNγ by the Treg cells, and IFNγ subsequently acts on nearby regulatory T cells to “destabilize” their suppressive phenotype. In contrast to the previously discussed examples which destabilize FoxP3 expression in Treg cells, a few cytokines binding to receptors that include the common γ-chain can enhance Treg cell proliferation and function. For instance, adding IL-2 enhances T cell proliferation, despite also stimulating Treg cells (87, 199).

Another possibility to be considered in cytokine-induced T cell resistance to Treg cells *in vitro* is proliferation and expansion of T cell quantity as the mechanism of Treg cell resistance, which should be distinguished from the ability to negate immunosuppressive signals. Especially in a murine *in vitro* system where Treg cell proliferation is limited, the capacity of T cells to proliferate may be independent of their ability to negate immunosuppressive signals by Treg cells. In other words, these T cells stimulated with cytokines may be equally susceptible to Treg cell-mediated suppression, but by increasing proliferation and quantity of T cells, the suppressive effect of Treg cells may become less apparent.

### Observations From Current Clinical Studies

One of the primary objectives of cancer immune therapy is to modulate anti-tumor T cell properties to reduce the tumor burden. However, the presence of immunoregulatory cells such as Treg cells are likely to interfere with the anti-tumor T cell response (9, 60, 216). Thus, overcoming the suppressive effects of Treg cells to potentially enhance anti-tumor T cell response in patients is a strategy currently under investigation. Many of the current clinical studies involve targeting surface receptors on Treg cells such as CD25, CTLA-4, and CCR4 (110, 124, 217).

However, clinical studies have not focused on rendering T cells resistant to the suppressive effects of Treg cells. Interestingly, some of the existing treatment methods may already foster T cells resistant to Treg cells. For instance, high dose IL-2 is part of the protocol for adoptive TIL therapy against metastatic melanoma, despite actively expanding immunosuppressive ICOS^+^ Treg cells (55, 218–221), supporting the possibility that high-dose IL-2 is successful because it may render TIL resistant to Treg cell suppression. Therefore, the dosage of systemic IL-2 administration in these studies may play an important role in promoting the T cell response against the tumor, since low dose IL-2 has been used to preferentially expand Treg cells to attenuate the progression of human autoimmune diseases (222, 223). To avoid IL-2-mediated expansion of immunosuppressive Treg cells, a pre-clinical study conducted by Charych et al. (224) suggested that NKTR-214, a biologic drug containing an IL-2 core conjugated to 6 releasable polyethylene glycol chains, can be utilized to preferentially induce IL-2 signaling on T cells while reducing the expansion of Treg cells. In this study, the ability of NKTR-214 to preferentially bind to IL-2Rβ over IL-2Rα induces a greater CD8^+^ T cell to Treg cell ratio, greater exposure to IL-2 in the tumor and a more robust anti-tumor immunity in comparison to aldesleukin. This particular approach is currently in clinical trials. Several other therapeutic strategies involving modified IL-2 biologics also suggest similarly promising results in their ability to preferentially enhance T cells over Treg cells (225, 226).

## Concluding Remarks

Regulatory T cells can be potent regulators of anti-tumor immunity, and numerous strategies have been proposed to reverse the suppressive effects of Treg cells. One promising approach involves rendering T cells resistant to the suppressive effects of Treg cells. Resistance to Treg cells can be achieved through modulation of intracellular molecules, co-stimulatory surface receptors or cytokines, all of which may act through partially redundant or overlapping mechanisms. Concepts discussed in this review primarily focus on strategies to manipulate the balance between T cells and Treg cells. However, future studies should validate these concepts in the context of anti-tumor immunity and focus on recapitulating many of these observations using primary human T cells.

## Author Contributions

All authors listed have made a substantial, direct and intellectual contribution to the work, and approved it for publication.

### Conflict of Interest Statement

The authors declare that the research was conducted in the absence of any commercial or financial relationships that could be construed as a potential conflict of interest.
